# Bee Sting-Inspired Inflammation-Responsive Microneedles for Periodontal Disease Treatment

**DOI:** 10.34133/research.0119

**Published:** 2023-04-18

**Authors:** Chuanhui Song, Xiaoxuan Zhang, Minhui Lu, Yuanjin Zhao

**Affiliations:** ^1^ Department of Rheumatology and Immunology, Institute of Translational Medicine, The Affiliated Drum Tower Hospital of Nanjing University Medical School, Nanjing 210002, China.; ^2^ State Key Laboratory of Bioelectronics, School of Biological Science and Medical Engineering, Southeast University, Nanjing 210096, China.; ^3^ Chemistry and Biomedicine Innovation Center, Nanjing University, Nanjing 210023, China.

## Abstract

Periodontal lesions are common and frustrating diseases that impact life quality. Efforts in this aspect aim at developing local drug delivery systems with better efficacy and less toxicity. Herein, inspired by the sting separation behavior of bees, we conduct novel reactive oxygen species (ROS)-responsive detachable microneedles (MNs) that carry antibiotic metronidazole (Met) for controllable periodontal drug delivery and periodontitis treatment. Benefiting from the needle-base separation ability, such MNs can penetrate through the healthy gingival to reach the gingival sulcus's bottom while offering minimal impact to oral function. Besides, as the drug-encapsulated cores were protected by poly (lactic-co-glycolic acid) (PLGA) shells in MNs, the surrounding normal gingival tissue is not affected by Met, resulting in excellent local biosafety. Additionally, with the ROS-responsive PLGA-thioketal-polyethylene glycol MN tips, they can be unlocked to release Met directly around the pathogen under the high ROS in the periodontitis sulcus, bringing about improved therapeutic effects. Based on these characteristics, the proposed bioinspired MNs show good therapeutic results in treating a rat model with periodontitis, implying their potential in periodontal disease.

## Introduction

Periodontitis is a common oral disease worldwide that brings great trouble to human health and life, being considered the leading cause of tooth loss in adults [1,2]. Generally, periodontitis is associated with anaerobic pathogens, among which the *Porphyromonas gingivalis* (P.g.) and *Fusobacterium nucleatum* (F.n.) are the primary culprits [3]. To defeat such a bacteria-induced plaque, antibiotic therapy is widely used, such as chlorhexidine [4], ornidazole [5], and metronidazole (Met) [6]. Among them, Met is a regular medication that deals with anaerobic bacteria and plays a role in periodontitis treatment [7]. Despite the demonstrated medical efficacy, systemic administration of Met may cause severe gastrointestinal adverse effects and allergy [8–10]. In addition, Met gargle used in clinics has poor drug retardation capacity and uncontrollable release, leading to drug overuse and unsatisfied therapeutic effects [11,12]. Thus, local drug delivery strategies toward periodontal diseases with high retention effects and inflammation responses are still highly desired.

**Fig. 1.**
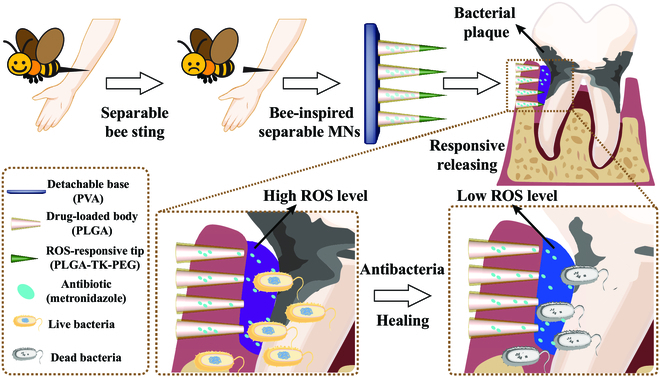
Device and manufacture of the bio-inspired MNs. The ROS-responsive MNs tips can be unlocked in the periodontitis status with high ROS levels, leading to increased drug concentration in the gingival sulcus.

Here, inspired by the sting separation behavior of bees, we propose detachable microneedles (MNs) with Met loading and inflammation-responsive ability to eliminate anaerobic bacteria and treat periodontal disease (Fig. 1). MNs, an emerging drug delivery technique, can penetrate through the skin in a painless, minimally invasive, and noninfective manner [13–20]. Besides, the solid matrix provided by the MNs has high drug loading potential and can be imparted with responsive release capacity after suitable modification [21–23]. Benefitting from these properties, drug delivery of MNs to diverse body parts, including the skin [24–26], heart [27–29], uterus [21,30], and blood vessels [31–33], has been widely explored. Although many achievements have been made [1], the application of MNs in oral disease is still rare, and how to keep them in the oral cavity for a long term without impacting normal oral function remains to be investigated. In addition, the drug release profiles of MNs, especially the responsive release, are waiting for further improvements.

In this paper, getting inspiration from the separable sting bees, we present the Met-loaded, reactive oxygen species (ROS)-responsive and detachable MNs to deliver drugs into the gingival sulcus for combating anaerobic bacteria to treat periodontitis. Poly (vinyl alcohol) (PVA) was chosen as the back of the MNs because it could promptly dissolve and detach from the tissue-penetrated MNs [13], thus reducing the impact on normal oral function. PLGA (poly (lactic-co-glycolic acid)) with the features of slow biodegradation and nontoxic ingredients was chosen as the shell of MNs to protect the inner drug core and avoid leakage of drugs to normal gingival tissue. Additionally, the ROS-responsive polymer (PLGA-thioketal-polyethylene glycol [PLGA-TK-PEG]) was employed to serve as MN tips, thus the MNs could respond to the high ROS condition among the infected gingival sulcus and directly release the Met into the gingival sulcus through the ROS-unlocked tips to conquer the P.g. and other pathogens. Based on these specialties, it was found that the bioinspired local drug delivery system showed an excellent therapeutic effect on periodontitis with wonderful biosafety in the rat model, indicating that the proposed MNs may offer a promising choice in periodontitis therapy and find new applications in other oral diseases.

## Results

**Fig. 2.**
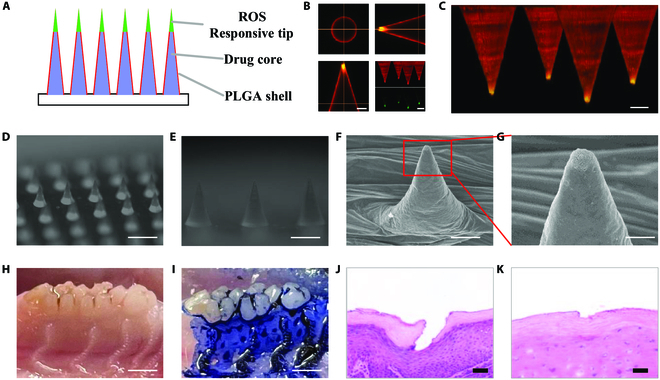
Characterization of MNs. (A) The illustrations of MNs’ composition. (B and C) The 3-dimensional reconstruction and cross-section of MNs with fluorescence showing the ROS-responsive tips in MNs. (D and E) The digital image of MNs. (F) The porous end of the tip after H_2_O_2_ incubation. (G) The enlarged image of (F). (H and I) The image of gingival before and after MN insertion. (J and K) The H&E staining of gingival after MN insertion immediately and after 5 d. Scale bars: 50 μm in (B) and (C), 1 mm in (D), 500 μm in (E), 100 μm in (F), 20 μm in (G), 1 mm in (H) and (I), and 20 μm in (J) and (K).

To fabricate the ROS-responsive MNs, we used 2-step casting to make the materials of each MN part different [34]. The thioketal bond in PLGA-TK-PEG could respond to ROS to achieve fast bond breaking (Fig. S1). ROS-responsive PLGA-TK-PEG was first added to negative MN molds, and the molds with solution were vacuumed to make ROS-responsive materials fill the end (Fig. 2A). The excess liquid was then removed with a pipette, and the solvent was evaporated to solidify the tips. After being cured completely, the PLGA solution was added gently to form the outer shell. Further, the solution was spiked with fluorescent nanoparticles and imaged using confocal microscopy to investigate the formation of tips. Figure 2B and C showed that the ends were green while the shells were red. After the solidification by solvent evaporation, we filled the body with Met in PLGA solution. We removed the excess material, after which the PVA solution was added as the base of the patch (Fig. 2D and E). The ROS could break the TK bonds based on redox reaction to act as the key to trigger drug release. To test the ROS-responsive property, the MNs were incubated with 1% H2O_2_ for 24 h, and the scanning electron microscopy showed that the tips were porous, indicating the ROS-responsive ability of MNs (Fig. 2F and G).

Compared with skin, the oral mucosa is softer to penetrate, which makes MNs convenient to apply. To test the mechanical specialties of the prepared MNs, the maximum applied forces of vertical direction were assessed using a manometer, during which the pressure could be recorded according to the move distance (Fig. S2A). The result showed that the mechanical strength of PLGA MNs was enhanced, consistent with the increase of PLGA concentration (Fig. S2B and C). Notably, the ROS-responsive mass had minimal effect on the mechanical property, owing to the comparability of PLGA and PLGA-TK-PEG. As the MNs with 15% PLGA could stand the compressive force of more than 0.3 N (the force to penetrate the skin) [13], such a concentration was decided to conduct the following in vivo tests.

During the in vivo experiments, an MN patch was applied to the oral cavity of an anesthetized rat. In rat oral cavity, the prepared MNs could easily penetrate the gingival and enter the gingival sulcus (Fig. 2H and I). In addition, the detachable base could be removed after application in the oral cavity leaving the drug-load body and ROS-responsive tip in the gingival, owing to the rapid dissolution of PVA when it encounters saliva (Fig. S3A and B). Furthermore, the buccal mucosa and tough could also be penetrated, indicating the wide application potential of MNs in oral disease (Fig. S3C and D). The safety of MN application should also be considered. For this purpose, the gingival tissue was collected after MN application. The hematoxylin and eosin (H&E) staining illustrated that the tissue could heal after MN removal, and a few inflammatory cell infiltrations were found in the tissue (Fig. 2J and K). All these data showed that MNs could be an ideal way to treat periodontal disease, as the periodontal-disease-causing bacteria accumulate in the bottom of the gingival sulcus. Notably, in the buccal mucosa and tongue body, the MNs also had excellent adhesive ability, indicating the potential of MNs for more oral application.

**Fig. 3.**
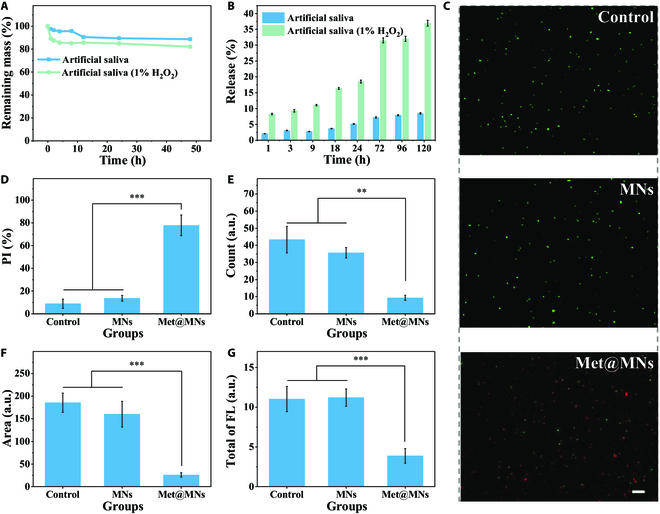
Drug release and antibacterial effect of MNs. (A) The remaining mass of MNs in artificial saliva with or without H_2_O_2_ under 37 °C. (B) The drug release profile of MNs in artificial saliva with or without H_2_O_2_ under 37 °C. (C) The Syto/PI staining of P.g. for live/dead test after different treatment. Scale bar: 20 μm. (D) The intensity quantity of (C). ***P < 0.001. (E) The clone number quantitative analysis of biofilm. **P < 0.01. (F) The staining area of biofilm. ***P < 0.001. (G) The quantitative analysis of the FISH staining of P.g. and F.n. in Fig. S4. a.u., arbitrary units. ***P < 0.001. FL, fluorescence.

As degradation is also a critical index of oral implant materials, we tested the degradation rate of MNs in artificial saliva. We detected the weight loss of MNs in artificial saliva with or without H_2_O_2_, and the results showed that although the weight loss was faster with H_2_O_2_ in the initial 8 h, the differences between each group were no longer obviously distinguishable thereafter (Fig. 3A). The ROS-induced quick degradation might contribute to the fast weight change, enabling the MNs to act as the ROS-responsive unlocking agent to release the inner drug. To test the drug release, we took fluorescein isothiocyanate-bovine serum albumin (FITC-BSA) as a mimic agent embedded in the core of MNs. After immersion in saliva for the selected time, the FITC-BSA in the supernatant was sampled and detected. The results showed that there was more drug release when H_2_O_2_ exited, indicating that the ROS unlocking ability could enhance drug release (Fig. 3B).

P.g. is the leading cause of periodontitis, so we tested the antibacterial toward P.g. The live/dead staining showed that the group after MN treatment exhibited minimal living bacteria compared with the control group without MNs (Fig. 3C and D). Besides, the F.n. is another pathogenic leading to periodontitis. We took the bacteria cocultured with MNs for 24 h to test the biofilm formation for evaluating the antibacterial effects of MNs toward pathogens. The biofilm staining showed that the drugs released from MNs could inhibit the biofilm from the dual bacteria (Fig. 3E and F and Fig. S4). Also, the fluorescence in situ hybridization (FISH) [35], with the ability to observe different bacteria, showed that both F.n. and P.g. had minimal fluorescence after MN treatment, which was consistent with the biofilm staining (Fig. 3G and Fig. S5).

**Fig. 4.**
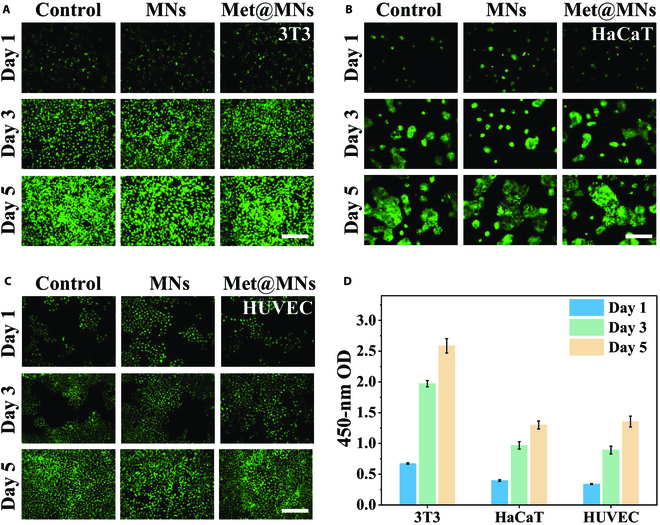
Biocompatibility of MNs. (A to C) The fluorescence images of (A) 3T3, (B) HaCaT, and (C) HUVECs incubated with MNs for different times. (D) The CCK-8 test of cell lines with MNs for different times. Scale bars: 100 μm. OD, optical density.

As the MN patch is directed to the diseased sites, the biocompatibility of the patch is crucially important during the treatment period. Besides the in vivo test, the fibroblast (3T3), oral mucosa cells (HaCaT), and human umbilical vein endothelial cells (HUVECs) were taken as regular cell lines to incubate with MNs for cell viability assessments. It was demonstrated by the calcein-acetoxymethyl staining that all the cells grew gradually and well whether they were cultured with MNs or not (Fig. 4A to C and Fig. S6). The Cell Counting Kit-8 (CCK-8) test further quantitatively indicated the excellent biocompatibility of MNs and the loaded antibiotic (Fig. 4D), suggesting reasonable design of our study, as the Met is effective toward the anaerobic bacteria. All those data showed that the MNs owned excellent antibacterial ability, which could be applied to oral disease and other infections.

**Fig. 5.**
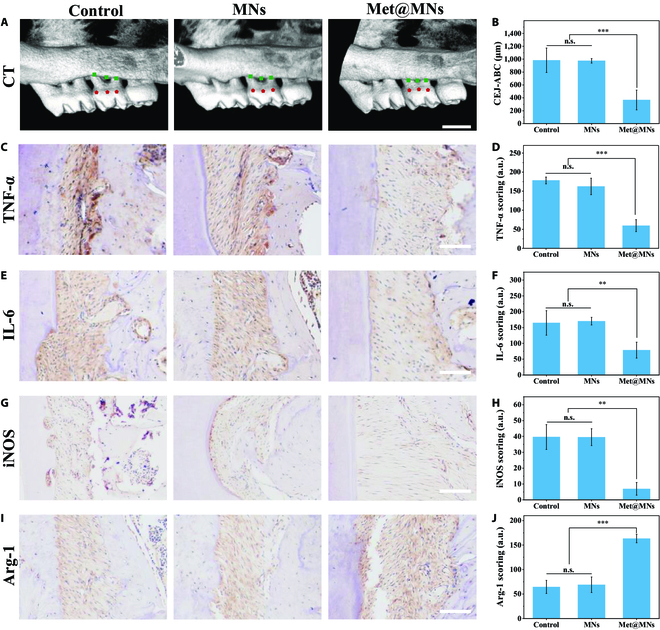
In vivo evaluations of anti-inflammatory performance in rat model. (A) The micro-CT of different treatments. (The squares and the pentagons show the measure points.) Scale bar: 1 mm. (B) The CEJ-ABC of teeth. ***P < 0.001. (C to J) The representative IHC staining images of periodontal tissue, including TNF-α (C), IL-6 (E), iNOS (G), and Arg-1 (I), as well as the quantitative analysis of IHC staining of TNF-α (D), IL-6 (F), iNOS (H), and Arg-1 (J). Scale bars: 100 μm. n.s., not significant. ***P < 0.001 and **P < 0.01.

The MN patch for periodontal treatment is rare but efficient, as the oral mucosa is easy to penetrate and adhere to. To verify the therapeutic effect of MNs, the periodontitis model was established in Sprague Dawley rats with tying silk thread. After 3 weeks of treatment, the micro-computed tomography (micro-CT) was used to detect the periodontal tissue. As in Fig. 5A, the MN treatment group showed minimal bone loss, while the empty MNs showed no noticeable change compared with the control group. The golden index to describe the periodontal condition was the length between the cementoenamel junction (CEJ) and alveolar bone crest (ABC) in the local jawbone. The measurement indicates that the treatment gained an excellent therapeutic effect, as the CEJ-ABC recovered to normal after treatment (Fig. 5B), attributing to the antibacterial effect on erosion of local pathogens, diminishing inflammation, and facilitating bone repair. Also, the relative inflammatory cytokines demonstrated the relief of local tissue since the tumor necrosis factor-α (TNF-α) and interleukin-6 (IL-6) were minor in the Met@MN group, compared with the control and empty MN groups through immunohistochemical (IHC) staining (Fig. 5C to F). The macrophage-relative cytokines also indicated the regional microenvironment trend to a recovery statement, the M2 macrophage-specific biomarker Arginase 1 (Arg-1) had a substantial elevation, and the M1 macrophage biomarker inducible nitric oxide synthase (iNOS) dramatically decreased after MN treatment (Fig. 5G to J). All data above proved that the bio-inspired MNs could act as an effective sterilization strategy to deal with periodontitis.

It is crucial to consider the biosafety of biomaterials, especially implanted biomaterials. To check the regional tissue after MN application, we collected the regional draining lymph node and the adjacent tissue, upper lip, and lower lip to check the status after application. The H&E staining results exhibited no noticeable pathological change after MN insertion with or without the drug (Fig. S7), indicating the excellent biocompatibility of the MNs. The inserted gingival also recovered to the normal state, which made it hard to distinguish visually or pathologically from the normal tissue (Fig. 2K). Furthermore, the major organs also showed no pathological change after the therapy period (Fig. S8). Also, the weights of rats during the whole period gradually increased and had no marked difference between groups (Fig. S9). All those data suggest the excellent biosafety of the MNs.

## Discussion

In summary, we have presented a bee-inspired Met-loaded separable MN that could ROS-responsively release drugs into the gingival sulcus to treat periodontists. To ensure proper oral function, drug-laden microcarriers like nanoplatform [36,37], spun scaffolds [38], injectable hydrogel [39], microspheres [40], and MNs [1] are initially showing promise in the treatment of periodontal diseases. Considering the continuous flow of saliva, most nanoparticles or microspheres cannot achieve long retention and controllable drug release, requiring repeated dosing. The injectable hydrogel and some hard scaffold need to implanted into the periodontal tissue with an invasive procedure, causing unpleasant feeling to the patient. The MN-based periodontal treatments have considerable potential, but further optimization of the MN design is needed to reduce the impact on oral function. Therefore, it is expected that still more efforts will be made to develop new detachable MNs to manage oral diseases.

Herein, inspired by the behavior of the bee sting separation, we have fabricated separable MNs with ideal responsive drug releasing and antiperiodontal pathogen abilities to conquer the above problems. Owing to the direct penetration of MNs into the sulcus, the drug could be injected into the bottom of the sulcus to kill the infiltrating bacteria. Also, the separation between MNs and the MN base minimized the effects of MNs on normal oral function as the space taken by MNs was reduced sharply. Besides, due to the slowly degraded protective PLGA shells and ROS-responsive PLGA-TK-PEG tips, these MNs achieved inflammation-responsive local drug delivery and thus effectively avoided the systemic adverse reactions caused by antianaerobic antibiotics. In addition, the MNs showed an outstanding performance in antibacteria and bone loss inhibition in the rat periodontitis model. These properties make the drug-loaded MNs potential candidates for periodontal diseases and other oral applications.

## Materials and Methods

### Reagent

Polyvinyl acetate, fetal bovine serum, and cell culture medium (high-glucose Dulbecco’s modified Eagle medium) were obtained from Adamas-beta (Shanghai, China). Fluorescent polystyrene nanoparticles (L2153 and L9777) were from Sigma. The CCK-8 was purchased from Bimake.

### Fabrication of MNs

The MNs were developed with a modified polydimethylsiloxane mold as previously described [41]. The tips of the MNs were designed with 220 μm (diameter) and 430 μm (height). The PLGA-TK-PEG (15%) in diglyme/water (95%/5%, v/v) was added to form ROS-responsive release tips. After a vacuum for 10 min, the solution entered the end of the tips, followed by solvent evaporation to solidify. PLGA (15%) was added to form the shells, the PLGA could attach to the model to form the shell after the solvent evaporates, and the space to form core could be retained. After total solidification, Met (10 mg/ml) dissolved in 8% polyvinyl acetate solution was put as the core of tips to act as the therapeutic. The corresponding fluorescent nanoparticles were added to observe MNs under fluorescence microscopy. To test the ROS responsiveness of MNs, 1% H_2_O_2_ was used to mimic the high ROS environment. After incubation for 24 h, a scanning electron microscopy test was conducted to analyze the change of tips.

### Mechanical strength tests

The force collection is launched when the sensor contacts the tips at a certain speed (0.2 mm/s). To test the MNs' penetration ability, MNs were pressed into a rat’s gingival. The tissue was dealt with gentian violet (1%) for 5 min to make the change obvious. After that, the tissue was collected for H&E staining.

### Characterization of MNs

To test the stability of MNs, a degradation detection was tested in simulated human saliva. The chips of MNs were weighed precisely, and after continuous vibration under 37 °C, the chips were dried and their mass was measured. The volume change was also detected by the measurement of MNs’ diameter and height.

### Drug releasing

The FITC-BSA was used to mimic the drug in the MNs. After storage in the artificial saliva for a predetermined time, the supernatant was taken out to detect the spectrum at 520 nm under the excitation of 494 nm. The concentration was calculated through the curve of the standard substance.

### Antibacterial test

P.g. is the leading pathogen resulting in the periodontists. To test the antibacterial capacity on P.g., the P.g. was seeded on a plate. The MNs were sterilized with ultraviolet irradiation for 24 hours. After MN application, bacteria were cultured for 7 d before checking the colony formation. Also, the MNs were incubated with bacteria in liquid nutrient medium for 24 h, after which the supernatants were recoated on a plate.

### Biocompatibility

The 3T3, HaCaT, and HUVEC cell lines were used as model cells to test the toxicity. The cells were added in a 96-well plate, with 3,000 cells per well, respectively. After attachment, the MNs were put into the culture system after sterilization. Then, the CCK-8 test and live/dead staining were conducted on the first, second, and third day. The calcein-acetoxymethyl/propidium iodide (PI) were added to detect the live/dead status following the manual with fluorescence microscopy.

### Treatment with MNs in rat models of periodontitis

All the animal experiments were under the approval of the Animal Investigation Ethics Committee of The Affiliated Drum Tower Hospital of Nanjing University Medical School (2021AE02007). For rat models of periodontitis, female Sprague Dawley rats were used. After anesthesia, silk sutures (4-0) were inserted and knotted around the subgingival area of the maxillary M2 (second molar). A high-sugar drink was applied during the model's build to accelerate the process. After confirming the model establishment with micro-CT, the silk was removed, followed by the treatment. The animal was divided into 3 groups: (a) control group (untreated); (b) empty MNs (without drug); and (c) MNs with drug. After the rat was anesthetized with 2% isoflurane, the MNs were inserted into the gingival sulcus through the gingival, horizontally, and the bases were removed when separating from the tips. The patches were checked daily to ensure that the MNs were in place. The MNs were reported when missing, and the rats' weights were measured every other day.

### Biosafety of MNs in vivo

The rats were sacrificed under anesthesia when the treatment was over. The major organs and local tissue in the oral cavity were collected for pathological examination. The tissues were fixed with 4% paraformaldehyde overnight, followed by embedment in paraffin. The maxillary bones were fixed in 4% paraformaldehyde. After micro-CT analysis, they were decalcified in a decalcifying solution (10% EDTA and 1% NaOH) for 20 d. After puncture confirmation of decalcification, tissue fixation and embedding were performed. The serial sections of rat maxillary were obtained along the mesiodistal direction, followed by H&E staining. IHC staining was conducted separately on maxillary sections with TNF-α, IL-6, iNOS, and Arg-1.

### Statistical analysis

All data in the figures were demonstrated as means with standard deviation (mean ± SD). The statistics were analyzed using GraphPad software by the Student *t* test between 2 groups and 1-way analysis of variance for more than 2 groups.

## Data Availability

The data that support the findings of this study are available from the corresponding author upon reasonable request.

## Supplementary Materials

Supplementary 1 Fig. S1. The chemical structural formula of PLGA-TK-PEG. Fig. S2. The loading force of MNs. Fig. S3. The biosafety of the MNs in vivo. Fig. S4. Digital picture of biofilm from P.g. and F.n. staining with crystal violet. Fig. S5. The FISH staining of P.g. and F.n. Fig. S6. The quantitative analysis of the cell counts in Fig. 4A to C. Fig. S7. The H&E staining of local tissue in the rat with different treatments. Fig. S8. The H&E staining of the heart, liver, spleen, lung, and kidney in different groups after treatment. Fig. S9. The body weight of rats in different groups after treatment.
