# Supplementary material for: Bee Sting-Inspired Inflammation-Responsive Microneedles for Periodontal Disease Treatment
Source: Research (Wash D C). 2023 Apr 18;6:0119. doi: 10.34133/research.0119 (PMC10202374; doi:10.34133/research.0119)
Supplement: Supplementary 1 — Fig. S1. The chemical structural formula of PLGA-TK-PEG. Fig. S2. The loading force of MNs. Fig. S3. The biosafety of the MNs in vivo. Fig. S4. Digital picture of biofilm from P.g. and F.n. staining with crystal violet. Fig. S5. The FISH staining of P.g. and F.n. Fig. S6. The quantitative analysis of the cell counts in Fig. 4A to C. Fig. S7. The H&E staining of local tissue in the rat with different treatments. Fig. S8. The H&E staining of the heart, liver, spleen, lung, and kidney in different groups after treatment. Fig. S9. The body weight of rats in different groups after treatment. [file research.0119.f1.docx]

**Supporting information**

**Title**

Bee sting-inspired inflammation-responsive microneedles for periodontal disease treatment

Microneedles for periodontal disease treatment

**Authors**

Chuanhui Song^1^, Xiaoxuan Zhang^2^, Minhui Lu^2^, Yuanjin Zhao^1,2,3*^

**Affiliations**

Chuanhui Song, Yuanjin Zhao^*^

Department of Rheumatology and Immunology, Institute of Translational Medicine, The Affiliated Drum Tower Hospital of Nanjing University Medical School, Nanjing, 210002, China.

Xiaoxuan Zhang, Minhui Lu, Yuanjin Zhao

State Key Laboratory of Bioelectronics, School of Biological Science and Medical Engineering, Southeast University, Nanjing 210096, China.

Yuanjin Zhao

Chemistry and Biomedicine Innovation Center, Nanjing University, Nanjing, 210023, China.

^*^Address correspondence to Yuanjin Zhao: yjzhao@seu.edu.cn


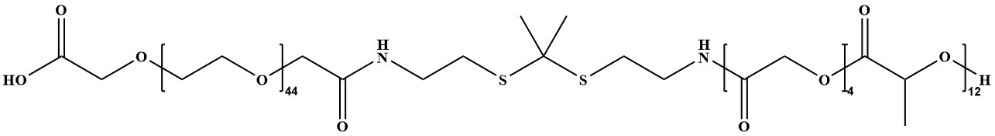


Figure S1. The chemical structural formula of PLGA-TK-PEG.


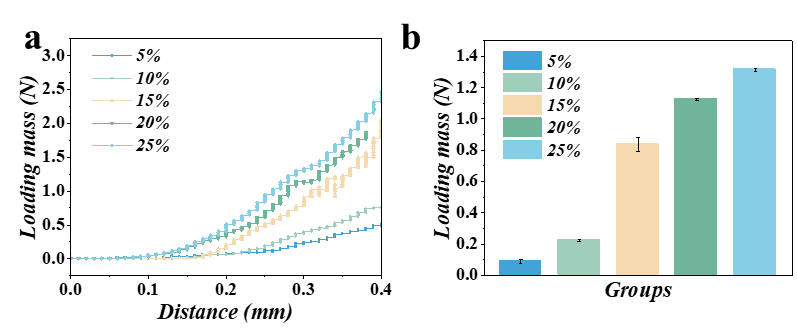


Figure S2. (a) The loading force curve of MNs from different concentrations of PLGA. (b) The max force of MNs.


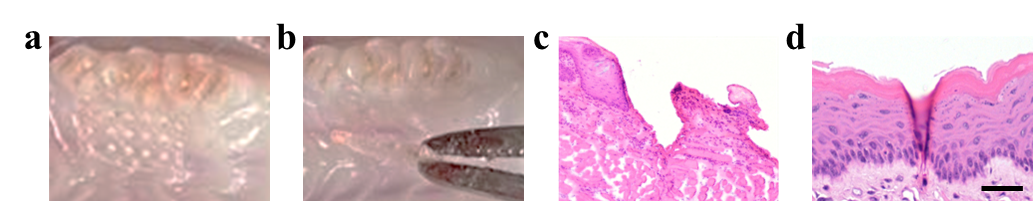


Figure S3. (a)The digital images of the detachable MNs apply in the rat oral cavity, (b) The detachable base can be removed. The H&E staining of buccal mucosa (a) and tough (b) after MNs apply. Scale bar=100 μm.


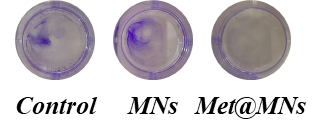


Figure S4. Digital picture of biofilm from P.g. and F.n. staining with crystal violet.


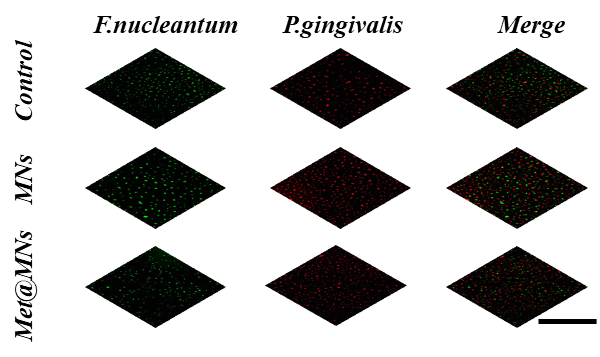


Figure S5. The FISH staining of P.g. and F.n. Scale bar=100 μm.


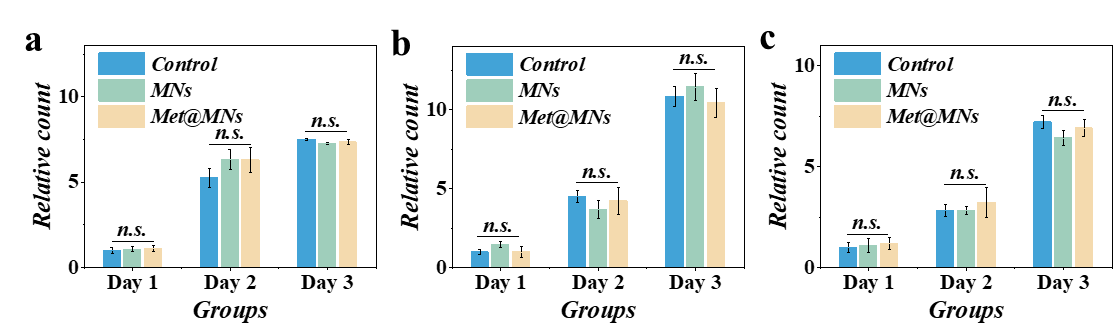


Figure S6. The quantitative analysis of the cell counts in Fig. 4 a-c. (a) 3T3; (b) HaCat; (c) HUVEC.


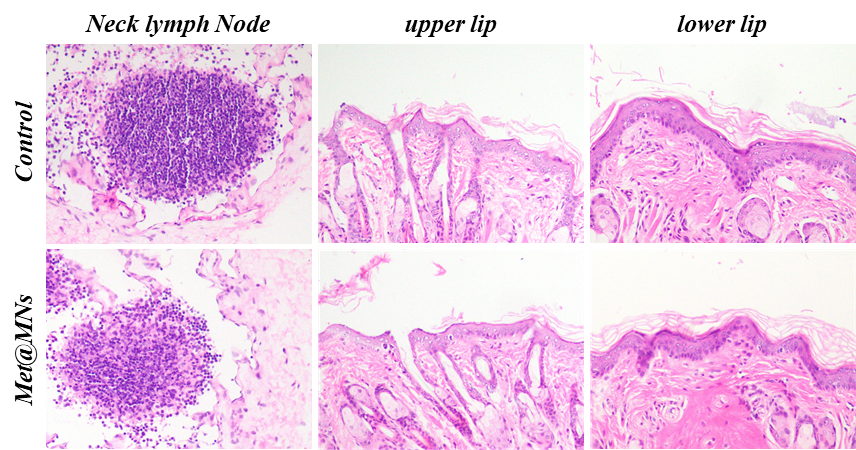


Figure S7. The H&E staining of local tissue in the rat with different treatments.


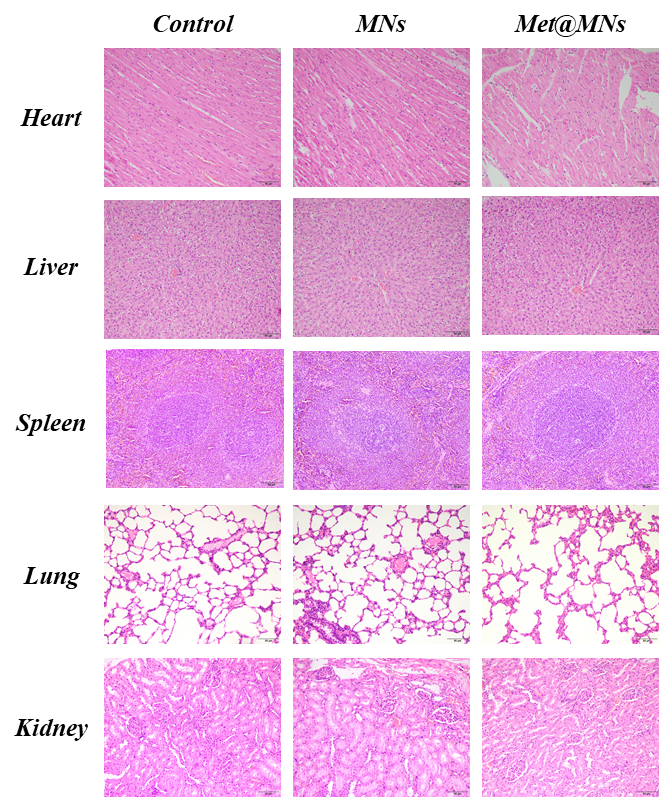


Figure S8. The H&E staining of the heart, liver, spleen, lung, and kidney in different groups after treatment.


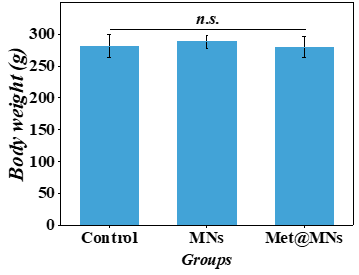


Figure S9. The body weight of rats in different groups after treatment.
